# A Reduction in Maximal Incremental Exercise Test Duration 48 h Post Downhill Run Is Associated with Muscle Damage Derived Exercise Induced Pain

**DOI:** 10.3389/fphys.2017.00135

**Published:** 2017-03-09

**Authors:** Bryna C. R. Chrismas, Lee Taylor, Jason C. Siegler, Adrian W. Midgley

**Affiliations:** ^1^Sport Science Program, College of Arts and Sciences, Qatar UniversityDoha, Qatar; ^2^ASPETAR, Qatar Orthopaedic and Sports Medicine Hospital, Athlete Health and Performance Research CentreDoha, Qatar; ^3^School of Sport, Exercise and Health Sciences, Loughborough UniversityLoughborough, UK; ^4^School of Science and Health, University of Western SydneySydney, NSW, Australia; ^5^Department of Sport and Physical Activity, Edge Hill UniversityOrmskirk, UK

**Keywords:** muscle soreness, maximum voluntary contraction, perception of effort, fatigue, exhaustion

## Abstract

**Purpose:** To examine whether exercise induced muscle damage (EIMD) and muscle soreness reduce treadmill maximal incremental exercise (MIE) test duration, and true maximal physiological performance as a consequence of exercise induced pain (EIP) and perceived effort.

**Methods:** Fifty (14 female), apparently healthy participants randomly allocated into a control group (CON, *n* = 10), or experimental group (EXP, *n* = 40) visited the laboratory a total of six times: visit 1 (familiarization), visit 2 (pre 1), visit 3 (pre 2), visit 4 (intervention), visit 5 (24 h post) and visit 6 (48 h post). Both groups performed identical testing during all visits, except during visit 4, where only EXP performed a 30 min downhill run and CON performed no exercise. During visits 2, 3, and 6 all participants performed MIE, and the following measurements were obtained: time to exhaustion (TTE), EIP, maximal oxygen consumption $\left(\overset{\cdot }{\text{V}}{\text{O}}_{2\text{max}}\right)$, rate of perceived exertion (RPE), maximum heart rate (HR_max_), maximum blood lactate (BLa_max_), and the contribution of pain to terminating the MIE (assessed using a questionnaire). Additionally during visits 1, 2, 3, 5, and 6 the following markers of EIMD were obtained: muscle soreness, maximum voluntary contraction (MVC), voluntary activation (VA), creatine kinase (CK).

**Results:** There were no significant differences (*p* ≥ 0.32) between any trials for any of the measures obtained during MIE for CON. In EXP, TTE decreased by 34 s (3%), from pre 2 to 48 h post (*p* < 0.001). There was a significant association between group (EXP, CON) and termination of the MIE due to “pain” during 48 h post (χ^2^ = 14.7, *p* = 0.002).

**Conclusion:** EIMD resulted in premature termination of a MIE test (decreased TTE), which was associated with EIP, MVC, and VA. The exact mechanisms responsible for this require further investigation.

## Introduction

Maximal incremental exercise (MIE) testing is utilized ubiquitously in research environments (Bassett and Howley, 2000), to provide a global overview of an individual's cardio-respiratory function (Astorino, 2009), which is of importance in both a clinical (Ingle, 2008) and an exercise physiology setting (Bentley et al., 2007). The nature of MIE requires participants to continue exercising until volitional exhaustion (Wagner, 2000), and consequently there is a high conscious and subconscious component to this test (Gibson et al., 1999). True maximal physiological variables may not be achieved during MIE if an individual fails to give a maximal effort (Taylor et al., 1955; Moffatt et al., 1994), and the termination of MIE is thought to be dependent on perceived effort (Gibson et al., 2003). Perception of effort is a complex interaction of feedforward-feedback mechanisms, and interpretation of afferent and efferent feedback. Subsequently any internal or external factor that could affect this feedback loop could influence perception of effort, and ultimately MIE test termination. One such factor is the physical condition of the participant upon arrival at the laboratory (McConnell, 1988). Disruption to an individual's physical condition could occur due to lack of sleep, fatigue and exercise induced muscle damage (EIMD).

Eccentric exercise (e.g., downhill running) causes preferential and increased disruption to type II muscle fibers (McHugh et al., 2000; Proske and Morgan, 2001) which is a known symptom of EIMD. Additionally, muscle fiber disruption within itself can activate mechano-sensitive fibers (i.e., group III/IV afferent fibers), which could increase perceived effort. Furthermore, this muscle fiber disruption can also increases noxious (e.g., bradykinin, prostaglandin, hydrogen ions) stimuli (Clarkson and Hubal, 2002), which could stimulate group III/IV metabo-sensitive afferent fibers responsible for feedback to the brain (Amann et al., 2010) and explain EIMD derived pain (i.e., delayed onset muscle soreness). This muscle soreness could be associated with an increased sense of effort during MIE exercise (Davies et al., 2011) as a result of an increase in motor unit recruitment following EIMD (Eston et al., 2000). Damage to selective fibers may require additional motor units to be recruited in order to achieve the same force output, which could increase an individual's perception of effort (Braun and Dutto, 2003). One study showed that EIMD had no effect on maximum rate of perceived exertion (RPE) despite a shorter TTE and subsequently a lower power output during cycling based MIE 48 h following eccentric squats (Davies et al., 2011). Similarly, increased ventilation was observed when EIMD signs and symptoms were present during MIE on a cycle ergometer, though no significant increase in perceived effort was shown (Yunoki et al., 2011). Yunoki et al. (2011) included both eccentric and concentric contractions (3 × 10 sets of leg press) 24 h prior to the MIE, compared to 100 eccentric squats utilized by Davies and colleagues, which may explain differential, though, similar experimental findings to Davies et al. (2011). Nevertheless, despite postulations from previous research that EIMD is likely to increase perceived effort during subsequent exercise, to date, no study has shown this. In fact, the aforementioned research suggests that there is no change in perceived effort.

Muscle soreness derived from EIMD could increase exercise induced pain (EIP), which is a measure of subjective “pain” experienced by an individual during exercise. However, the aforementioned studies only measured EIMD associated muscle soreness (i.e., using a visual analog scale) prior to the MIE test, for confirmation that EIMD had indeed occurred. The authors did not measure EIP (i.e., the pain experienced during the MIE test), which should not be confused with EIMD associated muscle soreness. Increased release of noxious substances and disruption to muscle fibers (Ellingson et al., 2014), could increase EIP via afferent feedback to the brain during exercise. EIP is likely to contribute to perceived effort (Mauger, 2014) and therefore, EIMD and associated muscle soreness could decrease performance in a MIE test due to increased perceived effort as a result of higher levels of EIP experienced during exercise. No previous study has examined the effect of EIP following EIMD on physiological performance during subsequent treadmill based MIE. Physiological differences (e.g., muscle recruitment, aerobic and anaerobic energy transfer) between cycling and running (Millet et al., 2009) may affect the relationship between perceived effort, EIP and treadmill based MIE outcome variables seen elsewhere (Davies et al., 2011; Yunoki et al., 2011). Given the important and multifaceted use of MIE derived outcome variables in research and in clinically and athletically focussed fields, it is essential that research investigates the effect of EIMD and muscle soreness on EIP and the perception of effort during MIE. One common method utilized within research to induce EIMD is a downhill run (Close et al., 2004; Cleary et al., 2006). Consequently, the novel aims of the present study were to (i) examine the effects of EIMD and muscle soreness on EIP and perception of effort during subsequent treadmill based MIE, (ii) explore the relationship between EIP, muscle soreness, EIMD and test duration in the MIE test, (iii) examine the effect of EIMD and muscle soreness on the physiological variables derived from treadmill MIE. It was hypothesized that the downhill run would result in a significant reduction in physiological performance and test duration in the MIE test and that this would be associated with increased EIP, with no change in perceived effort.

## Methods

### Participant characteristics

The fifty (14 female), apparently healthy participants who volunteered for this study had the following characteristics: [median (min - max)] age = 26 (18–49) y; mean (SD) height = 1.76 (0.09) m and mean (SD) mass = 70.7 (11.8) kg. Participants were free from musculoskeletal injury, non-smokers, and engaged in regular physical activity (> 30 min, three times a week for at least 6 months) and were familiar with treadmill running. Participants provided written informed consent, and were asked to adhere to written pre-measurement procedures for the duration of the study. These pre-measurement procedures stipulated that participants did not engage in any unaccustomed or high-intensity physical activity for 7 d prior to visit 1, that no large meals or stimulants were consumed within 4 h of each measurement, and that at least 500 ml of fluid was consumed 2 h prior to each measurement. Adherence to these procedures was monitored using a pre-measurement procedure checklist, which participants completed and signed prior to the commencement of each measurement. The apparent adherence was 100% in all instances. Test instructions and verbal encouragement were written down, and therefore, standardized for all trials, to ensure the investigator did not influence the results. Participants were free to leave the study at any point without reason, and anonymity, and confidentiality were ensured. Furthermore, all females completed the testing at the same phase of the menstrual cycle (follicular phase) to ensure differences in menstrual cycle did not affect the results. Ethical approval was granted by the University of Hull, Department of Sport & Exercise Science Ethics Committee, and all experimental procedures conformed to the Declaration of Helsinki, and National Institute of Health (NIH) standards for research with human participants.

### General experimental design

**Figure 1:**
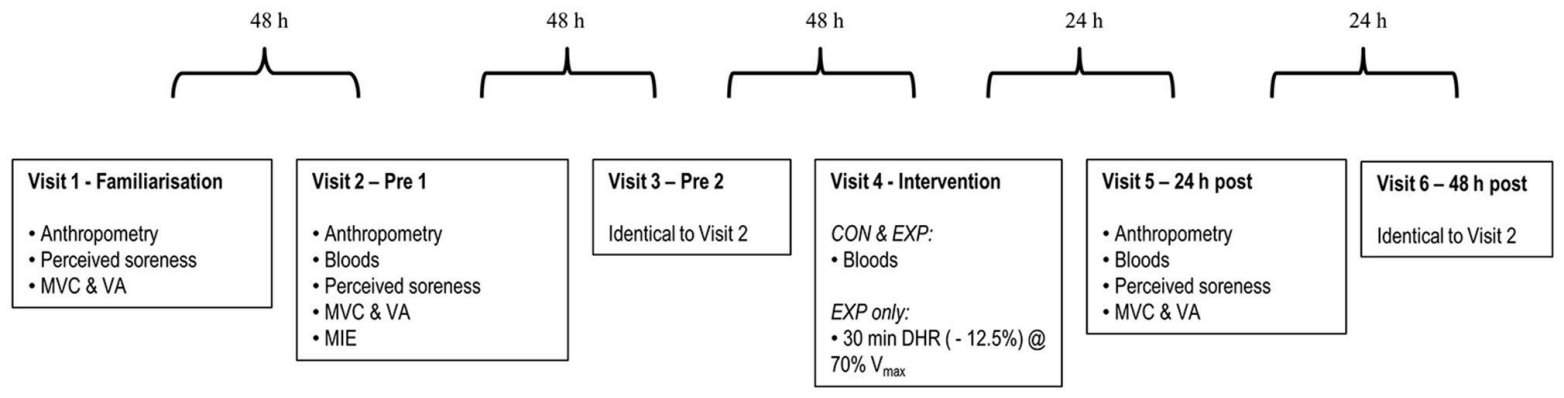
**Experimental schematic**. Measurements were obtained in the sequence listed for the control (CON) and experimental (EXP) groups. ROM, range of motion; CMJ, countermovement jump; SJ, squat jump; MVC, maximum voluntary contraction; VA, voluntary activation; MIE, maximal incremental exercise; DHR, downhill run; V_max_, maximal treadmill velocity.

Participants, randomly allocated into a control group (CON, *n* = 10, 3 female) or experimental group (EXP, *n* = 40, 11 female) visited the laboratory a total of six times: visit 1 (familiarization), visit 2 (pre 1), visit 3 (pre 2), visit 4 (intervention), visit 5 (24 h post) and visit 6 (48 h post). Both males and females were block randomized separately in to either group using the online software Randomizer (http://www.randomizer.org). Both groups performed identical testing during all visits, except during visit 4 (intervention), where EXP only performed a 30 min downhill run and CON performed no exercise (Figure 1). MIE was performed 48 h post intervention (visit 6) as this is the typical length of time recommended to abstain from exercise within pre-test guidelines for the majority of studies to attenuate any negative effects of muscle damage on the outcome variables. Testing times were held constant within individuals (± 1 h) to control for the confounding effects of circadian variation on exercise performance (Drust et al., 2005), and tests were performed in the order listed within the schematic (Figure 1). Data from the familiarization trial (visit 1) has not been included or used in any analyses as this trial was for familiarization purposes only.

Upon arrival to the laboratory tests were performed as described below. Times between each test were standardized within participants.

### Maximal incremental exercise tests

MIE was performed on a h/p/cosmos pulsar motorized treadmill (h/p/cosmos sports & medical gmbh, Nussdorf-Traunstein, Germany) at a gradient of 1% in order to reproduce the energetic cost of outdoor running on a flat surface (Jones and Doust, 1996). Initial treadmill velocity (median 6 km·h^−1^; range 4–9 km·h^−1^) was selected based on an estimated maximum treadmill velocity and/or the participants race time for 5 km, 10 km or a half marathon (if known), and replicated for all trials. If race time was unknown, initial starting velocity was set at 2 km·h^−1^ below the participants walk to run transition speed. These initial starting velocities were individualized in order to ensure participants completed the test within the recommended duration (Midgley et al., 2008). All participants completed a 5 min warm-up at the initial treadmill velocity before commencing the ramp protocol. Each stage of the ramp protocol was 1 min in duration and treadmill velocity increased by 0.1 km·h^−1^ every 6 s (i.e., 1 km·h^−1^ each stage) until volitional termination of the test. Terminal velocity was on average 13 km·h^−1^ (range 12–19 km·h^−1^). TTE was recorded as the total test duration (i.e., inclusive of the 5 min warm-up). Heart rate was measured continuously during all tests using short range radio telemetry. A Polar heart rate transmitter belt (Polar FS1, Polar Electro, OY, Finland) coated with electro-conductive gel (ECG gel, Meditec, Italy) to enhance signal detection, was fitted around the participants chest. For determination of HR_max_ the highest value obtained during the test was recorded. Additionally, throughout each test the rates of pulmonary oxygen uptake ($\overset{\cdot }{\text{V}}{\text{O}}_{2}$), and carbon dioxide output ($\overset{\cdot }{\text{V}}\text{C}{\text{O}}_{2}$) and minute ventilation (${\overset{\cdot }{\text{V}}}_{\text{E}}$) were measured continuously using an automated open circuit gas analysis system (Oxycon Pro, Jaegger, Hoechberg, Germany). Breath-by-breath data were reduced to 30 s stationary retrograde time average intervals and the highest averaged $\overset{\cdot }{\text{V}}{\text{O}}_{2}$,$\overset{\cdot }{\text{V}}\text{C}{\text{O}}_{2}$,${\overset{\cdot }{\text{V}}}_{\text{E}}$, and respiratory exchange ratio (RER) values attained during the incremental test were recorded. Additionally, maximum treadmill velocity (V_max_) was recorded. Following termination of the test BLa_max_ was measured immediately post using standard fingertip capillary blood sampling techniques. Capillary blood (~50 μL) was collected into a heparin coated plastic capillary tube (Radiometer Ltd, West Sussex, UK) and immediately analyzed using an ABL77 blood gas analyser (Radiometer, West Sussex, UK). Intra-assay CV for duplicate samples was 1.8%.

#### Measurement of psycho-physiological variables

Ratings of perceived exertion (RPE) (Borg 6–20 scale), and EIP (Cook et al., 1997) were assessed during MIE. The EIP scale is a category scale with ratio properties. The authors provide the following information and instructions for use of the EIP scale. The scale ranges from 0 (no pain at all) to 10 (extremely intense pain, almost unbearable). If the subjective intensity increases above 10 the participant is free to choose any number larger in proportion to 10 that describes the proportionate growth of the sensation (Cook et al., 1997). Prior to each test the participants were provided with standardized verbal and written instructions for each scale, which were replicated for each trial. During the last 15 s of each incremental phase of the MIE (i.e., the last minute) a value for RPE and EIP was obtained in a random order. Only the maximum values for both RPE and EIP obtained during the MIE test are reported in the results.

#### Reasons for termination

Following completion of each MIE test, participants were asked to complete a self-designed questionnaire relating to the contributory factors to the termination of the test. This questionnaire consisted of 16 contributory factors, and an “other” factor box where participants could specify a factor if not listed. The 16 contributory factors included pain, overall exhaustion, discomfort, nausea, boredom, lack of motivation and concerns about injury. Participants had to provide an answer for each factor by ticking one box only, which was either “not a contributory factor,” “a minor contributory factor,” “a major contributory factor,” or “the only contributory factor.”

### Downhill run protocol

Participants in EXP performed a 30 min downhill run (-12.5% grade) at 70% of their V_max_ determined by averaging the maximal treadmill velocity from visits 2 and 3. This protocol was employed as previous research employing a similar downhill run protocol reported signs and symptoms associated with EIMD (Close et al., 2004; Cleary et al., 2006).

### Anthropometry

Body mass (kg) and height (m) were measured using SECA balance scales (Vogel & Halke, Hamburg, Germany) and a wall-mounted Holtain Stadiometer (Holtain Ltd., Crymych, Dyfed) respectively.

### Blood collection and analyses

Fingertip capillary blood samples were collected using standard techniques following 10 min rest in a supine position, at the time points shown in Figure 1 for the determination of plasma creatine kinase (CK), hemoglobin (Hb) and haematocrit (Hct).

Plasma CK was determined from a 32 μl fingertip capillary blood sample while participants were semi-recumbent on a treatment couch. The sample of whole blood was immediately pipetted to a test strip and analyzed for CK using a colorimetric assay procedure (Reflotron, Boehringer Mannheim, Germany). The intra-assay coefficient of variation (CV) for this was 9.0%.

For the analysis of Hb and Hct all samples were measured in triplicate and averaged. In order to determine Hct concentration, blood was collected into sodium heparinised microhaematocrit capillary tubes (Hawksley, Lancing, Sussex, UK) before being centrifuged in a Hawksley HaematoSpin 1400 (Hawksley, Lancing, Sussex, UK) for 2 min at 14,000 G. Subsequently, capillary tubes were placed on a Hawskley microhaematocrit tube reader (Hawksley, Lancing, Sussex, UK) and the Hct (%) recorded. The intra-assay CV was 3.2%. For Hb determination, 10 μl of blood was collected onto a HemoCue® Hb 201 microcuvette (HemoCue® Ltd., Dronfield, Derbyshire, UK) and placed into a HemoCue® Hb 201^+^ (HemoCue® Ltd., Dronfield, Derbyshire, UK) in accordance with manufacturer's instructions. The intra-assay CV for this procedure was 1.2%. Hb and Hct concentrations were used to measure changes in plasma volume according to the Dill and Costill method using the following equation (Dill and Costill, 1974):

(1) $$\begin{array}{l} ((100*(Hbpre/Hbpost))*((1-(Hctpost-100))/\\ \;(1-(Hctpre-100)))-100 \end{array}$$

CK measures were subsequently adjusted to account for shifts in plasma volume. The percentage change in plasma volume was either added or subtracted from the concentration of CK as required.

### Assessment of muscle soreness

Perceived muscle soreness was measured using a visual analog scale consisting of a 100 mm horizontal line which ranged from 0 mm (no soreness) to 100 mm (unbearable soreness) in order to confirm if the downhill run induced EIMD. The soreness quantification was determined by measuring the distance from the left edge of the 100 mm line to the marked point in millimeters and this value was used for the analysis. Muscle soreness was assessed during 5 min of treadmill running at a speed of 0.5 km·h^−1^ above each participant's walk-to-run transition speed (preferred transition speed). Soreness measurements were obtained at the end of the first minute (Chrismas et al., 2017). The 5 min treadmill run also served as a warm-up. Preferred transition speed was determined during the first visit and used for all subsequent tests. Initially participants started walking at 2 km.h^−1^. Velocity was increased by 0.1 km.h^−1^ every 6 s and participants were instructed to transition from a walk to a run when they felt comfortable doing so. This was also verbally confirmed by each participant, and repeated three times, and an average taken.

### Maximum voluntary contraction

Bilateral MVC of the quadriceps was assessed using a Biodex isokinetic dynamometer (Biodex System 3, Biodex Medical Systems, Inc., Shirley, NY). Previous research has demonstrated that maximum voluntary peak torque is highly reproducible (TE ≤ 6.1%, ICC ≥ 0.85) in physical active individuals using a Biodex System 3 (Almosnino et al., 2011). Participants were seated comfortably on the adjustable chair of the dynamometer with hip flexion at 85°. The chair position was modified until the lateral femoral condyle was aligned with the axis of rotation of the dynamometer. Seat length and height were recorded for each participant and replicated during subsequent testing. Restraining straps were fixed across the chest, pelvis, and thigh of the exercising leg to prevent any extraneous movement. The exercising leg was secured to the dynamometer arm by attaching the ankle cuff proximal to the lateral malleolus. Range of motion and gravity corrections for limb mass were performed before each assessment. Participants always performed three MVC separated by a 3 min rest, in an attempt to reduce the effects of fatigue (Newman et al., 2003). Testing of the right leg always preceded that of the left leg. Participants were instructed to keep their hands crossed in front of their chest during each contraction. Bilateral isometric MVC of the knee extensors was assessed at an angle of 70° in accordance with previously published research (Christou et al., 2002; Howatson and Milak, 2009). Each MVC lasted for 5–6 s. The peak of the three MVCs was used for analysis, determined from the highest point of the peak torque curve.

During each isometric MVC VA was measured using electrical stimulation. Twitches were evoked using a percutaneous neuromuscular electrical stimulator (Digitimer model DS7AH, Welwyn Garden City, UK) applied to the quadriceps muscles via two (8 × 10 cm) carbon rubber moistened surface electrodes (Platimum 895340, PALS, Axelgaard, CA, USA) positioned proximally over the vastus lateralis, and distally over the vastus medialis. This method of electrical stimulation was chosen as it has been suggested that percutaneous electrical stimulation (single or paired) provides a similar force to nerve stimulation in either the fatigued or non-fatigued quadriceps muscle (Verges et al., 2009). A permanent pen was used to outline the position of each electrode, so as to minimize variability in electrode placement between repeated testing. Electrodes were secured in place by Velcro. Initially, maximal current intensity was determined by stimulating the quadriceps with an electrical impulse (200 μs, 400 V) of increasing current steps of 50 mA, until a plateau in torque was observed. The current was further increased by 50 mA to ensure maximal activation. During each MVC a superimposed twitch was evoked once the participant had reached maximum torque and a plateau was observed. The resting twitch was imposed approximately 3 s after the contraction. Muscle activation was calculated using the interpolated twitch technique (ITT) from the superimposed and resting twitch using the following equation: ITT (%) = [1 – size of interpolated twitch/size of resting twitch] × 100. The ITT was chosen over the central activation ratio, as previous findings reported that the central activation ratio overestimates VA and is not sensitive enough to detect minor fluctuations in voluntary force (Morton et al., 2005).

### Statistical analyses

The number of participants required for this study was determined *a priori* with an alpha level of 0.05 using a 2 tailed *t*-test for the main outcome measures of TTE and muscle soreness using Power Analysis and Sample Size Software (PASS) version 13.0 (NCSS, LLC, Utah, USA). Group sample sizes of 10 for the control group, and 40 for the experimental group, achieved 99 and 94% power to detect minimum worthwhile effects of 30 s (TTE) and 15 mm (muscle soreness) respectively. Analyses were completed using the statistical software package IBM SPSS Statistics version 19.0 (SPSS Inc, Chicago, IL, USA) and graphs created using SigmaPlot version 12.3 (Systat Software Inc, CA, USA). For descriptive purposes the mean and standard deviation have been used to report the central tendency and dispersion of the observed data where normally distributed, and the median and range were used where not normally distributed.

Data obtained in visit 2 (pre 1) and visit 3 (pre 2) were used for reproducibility analyses. Combinations of statistical methods were chosen in order to compare reproducibility between different measures and different studies. First systematic bias was tested using two-tailed dependent *t*-tests. Absolute measurement error was determined using repeated measures CV. The CV (expressed as a percentage) was calculated by dividing the standard deviation of the differences by the square root of two and dividing the answer by the grand mean (Hopkins, 2000). Relative reliability was determined using a two-way random model intraclass correlation coefficient (ICC), which is a measure of the ratio of between-subject variance to within-subject variance.

Only the data obtained from visit 3 (pre 2) visit 4 (intervention), visit 5 (24 h post) and visit 6 (48 h post) were used for subsequent experimental analyses. Independent *t*-tests were used to check there were no significant differences in visit 3 (pre 2) between the control group and experimental group. Linear mixed models were chosen to determine if there were any differences in the dependent variables between CON and EXP across trials. This type of analysis was preferred as it allows for missing data and can specify different covariate structures for repeated measures data. First fixed and random factors for the linear mixed model were fit for each dependent variable and the main effects for trial, group and the interaction effect (trial x group) were analyzed by plotting the mean values. The most appropriate model was chosen using the likelihood ratio test. This method uses the χ^2^ critical test statistic to decide which model is the best fit based on the change in the −2 restricted log likelihood of two nested models. Second, normality and homogeneity of variance of the residuals were checked using quantile-quantile plots and scatter plots respectively, and deemed plausible in each instance. Pearson's r was used to examine the relationship of TTE during visit 6 (48 h post) with EIP, soreness, VA, and MVC during visit 5 (24 h post) and visit 6 (48 h post). Chi square analysis was used to investigate the association between group (EXP and CON) and termination of the MIE due to “pain” during visit 6 (48 h post). Data were analyzed separately by sex to investigate whether there were any significant differences between males and females. However, as there were no significant differences between males and females (*p* ≥ 0.23) the data set was collapsed for the final analyses. Additionally, age was entered into the model as a covariate, but as this did not make any significant difference it was removed for the final analyses to increase statistical power, and satisfy the principal of parsimony. One participants data for RPE was removed from analysis as it was unusually low throughout all trials (10, 11). However, it was clear from this participants physiological data (e.g., $\overset{\cdot }{\text{V}}{\text{O}}_{2}$,$\;\overset{\cdot }{\text{V}}\text{C}{\text{O}}_{2}$,$\;{\overset{\cdot }{\text{V}}}_{\text{E}}$ etc.) that a maximal effort was provided. Nevertheless, despite familiarization, this participant was clearly unable to convey their true perception of effort, and subsequently their RPE data has been omitted. The two-tailed alpha level for significance testing was set as *p* ≤ 0.05.

## Results

### Reproducibility

The mean responses to the indirect markers of EIMD and MIE completed during visit 2 (pre 1) and visit 3 (pre 2) are shown in Table 1. Dependent *t*-tests indicated there was no systematic bias (*p* = 0.05) between trials for any of the variables (Table 1).

**Table 1:** **Values obtained during visit 2 (pre 1) and visit 3 (pre 2) for the indirect markers of EIMD tests and the MIE tests completed for CON and EXP (*n* = 50) combined. Values are reported as mean (SD)**.

**Measure**	**Visit 2**			**Visit 3**			**Visit 2–Visit 3 differences**			
	**Mean**	**SD**	**Range**	**Mean**	**SD**	**Range**	**Mean diff**	**95% CI**	**S_d_**	***p*-value**
*EIP*_max_	1.8	1.2	0–4	2.0	1.2	0–4	0.2	−0.02, 0.4	0.6	0.07
TTE (s)	998	86	798–1210	1000	92	784–1229	2	−2.5, 5.4	14.0	0.48
Soreness (mm)	11	12	0–38	10	11	0–34	−1	−2.6, 0.9	6.0	0.35
CK (IU/L)	109.8	40.4	42.6–283.0	108.7	42.7	41.3–210.0	−1.0	−7.9, 10.0	31.6	0.82
MVC dom (N.m)	158.9	49.5	72.6–254.7	155.1	47.3	62.8–253.0	−3.7	−9.3, 1.9	18.2	0.19
MVC non-dom (N.m)	148.7	46.3	64.5–226.6	147.4	46.0	58.4–226.9	−1.3	−6.8, 4.1	17.8	0.62
VA dom (%)	83	10	67–97	86	8	68–96	3	−0.3, 5.4	9.2	0.07
VA non-dom (%)	83	11	66–97	84	9	63–96	1	−1.8, 4.3	10.0	0.42
$\overset{\cdot }{\text{V}}{\text{O}}_{2\text{max}}$ (L·min^−1^)	3.09	0.71	1.76–4.21	3.13	0.72	1.78–4.20	0.04	−0.04, 0.1	0.24	0.30
$\overset{\cdot }{\text{V}}\text{C}{\text{O}}_{2\text{max}}$ (L·min^−1^)	3.60	0.87	2.02–4.85	3.64	0.82	1.53–5.33	0.04	−0.06, 0.15	0.3	0.43
V_Emax_ (L·min^−1^)	112	27	61–165	113	25	64–157	0	−2.5, 3.1	9.0	0.85
VT (km·h^−1^)	11.1	1.5	7.3–14.3	11.2	1.6	7.0–14.5	0.1	−0.1, 0.4	0.86	0.32
HR_max_ (b·min^−1^)	196	9	176–218	195	9	171–216	−1	−1.5, 0.3	3.0	0.21
BLa_max_ (mmol/L)	11.4	2.8	5.5–16.0	11.3	2.9	5.5–16.0	−0.1	−0.3, 0.2	0.9	0.67
RER_max_	1.17	0.07	1.01–1.34	1.17	0.06	1.01–1.31	0.01	−0.02, 0.02	0.1	0.94
RPE_max_	19	2	17–20	19	2	17–20	0	−0.2, 0.2	0.5	0.89

*Any discrepancies between the mean diff and means is due to rounding error; EIMD, exercise induced muscle damage; MIE, maximal incremental exercise; CON, control; EXP, experimental; SD, between subject standard deviation; mean diff, mean difference; 95% CI, lower and upper bounds of the 95% confidence interval for the mean difference; S_d_, within-subject standard deviation; CK, creatine kinase; MVC, maximum voluntary contraction; dom, dominant; non-dom, non-dominant; VA, voluntary activation; $\overset{\cdot }{V}O_{2max}$, maximum oxygen consumption; TTE, time to exhaustion (total test duration inclusive of 5 min warm-up); $\overset{\cdot }{V}CO_{2max}$, maximum velocity of carbon dioxide; V_Emax_, maximum ventilation; VT, ventilatory threshold; HR_max_, maximum heart rate; RER_max_, maximum respiratory exchange ratio; BLa_max_, maximum blood lactate concentration; RPE, maximum rating of perceived exertion (i.e., at exhaustion), EIP, exercise induced pain*.

The CV, and ICC for the indirect markers of EIMD and MIE are reported in Table 2. Reproducibility of the measurements employed in the present study is shown for quality assurance purposes only.

**Table 2:** **Reproducibility statistics for the indirect markers of EIMD and the MIE outcomes (*n* = 50)**.

**Measure**	**CV (%)**	**ICC**
EIP_max_	23.3	0.82
TTE (s)	1.9	0.98
Soreness (mm)	8.0	0.89
CK (IU/L)	20.0	0.90
MVC dom (N.m)	8.2	0.93
MVC non-dom (N.m)	8.5	0.93
VA dom (%)	7.7	0.79
VA non-dom (%)	8.5	0.82
$\overset{\cdot }{\text{V}}{\text{O}}_{2\text{max}}$ (L·min^−1^)	5.5	0.94
$\overset{\cdot }{\text{V}}\text{C}{\text{O}}_{2\text{max}}$ (L·min^−1^)	6.2	0.93
V_Emax_ (L·min^−1^)	5.4	0.95
VT (km·h^−1^)	3.8	0.85
HR_max_ (b·min^−1^)	1.1	0.94
BLa_max_ (mmol/L)	5.6	0.95
RER_max_	3.6	0.78
RPE_max_	4.9	0.88

*EIMD, exercise induced muscle damage; MIE, maximal incremental exercise; CV, coefficient of variation for repeated measures; ICC, intraclass correlation coefficient (two-way random model for a single rater); range ICC's = 0.78–0.98. See footnotes of Table 1 for definitions of the other abbreviations not listed*.

### Maximal incremental exercise test results

There were no significant differences (*p* ≥ 0.32) between visit 3 (pre 2) and visit 6 (48 h post) for any of the measures obtained during MIE for CON (Table 3).

**Table 3:** **Values obtained during visit 3 (pre 2) and visit 6 (48 h post) for the indirect markers of EIMD tests and the MIE tests completed for CON and EXP (*n* = 50)**.

**Measure**	**CON (*n* = 10)**				**EXP (*n* = 40)**			
	**Visit 3**	**Visit 6**	**Mean diff**	**95% CI**	**Visit 3**	**Visit 6**	**Mean diff**	**95% CI**
EIP_max_ [median,(min – max)]	2 (0–3)	1 (0–3)	0	−1.3, 0.8	2 (0–4)	4 (0–7)^a+b^	2	1.4, 2.5
TTE (s)	997 (90)	1003 (83)	6	−18.4, 31.4	997 (94)	964 (98)^a+b^	−34	−45.2, −19.7
Soreness (mm)	8 (5)	11 (14)	3	−18, 10	11 (12)	62 (21)^a+b^	51	44, 58
CK (IU/L)	112 (44)	116 (109)	4	−132, 142	116 (80)	227 (131)^a+b^	111	42, 179
MVC dom (N.m)	158 (68)	157 (60)	−1	−9, 11	156 (42)	142 (49)^a+b^	−14	−19, −9
MVC non−dom (N.m)	153 (62)	145 (59)	−8	−19, 3	145 (42)	131 (44)^a+b^	−14	−19, −8
VA dom (%)	85 (9)	86 (8)	1	−8, 5	86 (8)	81 (13)^a+b^	−5	−8, −2
VA non-dom (%)	88 (7)	86 (8)	−2	−6, 8	83 (9)	78 (11)^a+b^	−5	−9, −2
$\overset{\cdot }{\text{V}}{\text{O}}_{2\text{max}}$ (L·min^−1^)	3.20 (0.84)	3.07 (0.77)	−0.13	−0.41, 0.16	3.08 (0.74)	3.00 (0.77)	−0.09	−0.21, 0.04
$\overset{\cdot }{\text{V}}\text{C}{\text{O}}_{2\text{max}}$ (L·min^−1^)	3.73 (0.98)	3.82 (0.98)	0.09	−0.31, 0.48	3.53 (0.57)	3.43 (0.54)	−0.09	−0.89, 0.10
${\overset{\cdot }{\text{V}}}_{\text{Emax }}$ (L·min^−1^)	120 (34)	118 (35)	−2	−13.5, 8.4	107 (24)	108 (25)	1	−6.0, 4.7
VT (km·h·^−1^)	11.1 (1.0)	10.9 (0.8)	−0.2	−2.2, 1.7	11.2 (1.7)	11.0 (1.5)	−0.2	−1.1, 0.7
HR_max_ (b·min^−1^)	194 (7)	194 (7)	0	−4.2, 4.7	195 (9)	190 (11)^a+b^	−5	−6.8, −2.4
BLa_max_ (mmol/L)	12.3 (3.2)	12.2 (3.5)	−0.1	−1.5, 1.3	10.5 (2.9)	9.6 (2.7)^a+b^	−0.9	−1.6, −0.2
RER_max_	1.17 (0.06)	1.21(0.07)	0.04	−0.01, 0.08	1.16 (0.07)	1.16 (0.08)	0	−0.03, 0.02
RPE_max_ [median,(min – max)]	19 (17–20)	19 (17–19)	0	−0.3, 0.2	19 (17–20)	19 (17–20)	0	−0.1, 0.3

*Values are reported as mean (SD) unless otherwise specified*.

a+b *significant interaction effect (group x trial). MIE, maximal incremental exercise; EIMD, exercise induced muscle damage; CON, control; EXP, experimental. See footnote of Table 1 for definitions of the abbreviations and lettering not listed*.

In EXP there was a significant group x trial interaction effect (*F* = 7.9, *p* = 0.007) for TTE. *Post-hoc* comparisons revealed a mean decrease of 34 s (3%), from pre 2 to 48 h post (*p* < 0.001). Furthermore, there was a significant group x trial interaction effect for BLa_max_ (*F* = 5.0, *p* = 0.03), and HR_max_ (*F* = 3.8, *p* = 0.04). *Post-hoc* comparisons revealed a mean decrease of 0.9 mmol/L (9%) for BLa_max_ (*p* < 0.001), and 5 b.min^−1^ (3%) for HR_max_ (*p* < 0.001) from pre 2 to 48 h post (Table 3). In addition there was a significant group x trial interaction effect (*F* = 13.3, *p* = 0.001) for perceived pain. *Post-hoc* comparisons revealed a mean increase of 100%, from pre 2 to 48 h post (*p* < 0.001). No statistically significant differences (*p* = 0.08) between pre 2 to 48 h post were observed in the experimental group for any of the other measures obtained (Table 3).

### Indirect markers of exercise induced muscle damage

In EXP there was a significant group x trial interaction effect (*F* = 4.5, *p* < 0.01) for CK. *Post-hoc* comparisons (*p* < 0.001) revealed a mean increase of 111.0 IU/L (96%) from pre 2 to 48 h post (Table 3). Additionally, there was a significant group x trial interaction effect (*F* = 34.8, *p* < 0.001) for muscle soreness. *Post-hoc* comparisons (*p* < 0.001) demonstrated a mean increase of 51 mm from pre 2 to 48 h post (Table 3). Furthermore, there was a significant group x trial interaction effect for MVC in the dominant leg (*F* = 9.5, *p* < 0.001), and non-dominant leg (*F* = 7.5, *p* = 0.001). *Post-hoc* comparisons revealed a mean decrease of 12.2 N.m (8%) in the dominant leg (*p* < 0.001), and 11.6 N.m (8%) in the non-dominant leg (*p* < 0.001) from pre 2 to 48 h post (Table 3). There was a significant group x trial interaction effect for VA in the dominant leg (*F* = 3.9, *p* = 0.02), and non-dominant leg (*F* = 4.8, *p* = 0.03). *Post-hoc* comparisons revealed a mean decrease of 8% in the dominant leg (*p* < 0.001), and 6% in the non-dominant leg (*p* < 0.001) from pre 2 to 48 h post (Table 3). There were no significant differences between any trials (*p* = 0.51) for any of the markers of EIMD in CON (Table 3).

### Pearson's r

There was a significant relationship between EIP, MVC, and VA with TTE during visit 6 (48 h post) (*p* = 0.05) please see Table 4.

**Table 4:** **Relationship between TTE, EIP, MVC, VA, and soreness during visit 6 (48 h post) analyzed using Pearson's r (*n* = 50)**.

	**Soreness**	**VA**	**MVC**	**EIP_max_**
TTE	*r* = −0.16, *p* = 0.26	*r* = 0.32, *p* = 0.04^*^	*r* = 0.36, *p* = 0.02^*^	*r* = 0.29, *p* = 0.05^*^

See footnote of Table 1 for definitions of the abbreviations;

* *statistically significant relationship*.

### Chi square

There was a significant association between group (EXP, CON) and the contribution of “pain” to termination of the MIE during visit 6 (48 h post) (χ^2^ = 14.7, *p* = 0.002). In the CON group 60% of the participants said pain was “not a contributory factor” to them terminating the test, 30% reported pain as “a minor contributory factor,” 10% “a major contributory factor,” and 0% “the only contributory factor” to them terminating the MIE test. In the EXP group 13% reported that pain was “not a contributory factor,” 13% reported pain as “a minor contributory factor,” 72% “a major contributory factor,” and 3% reported pain as “the only contributory factor” to them terminating the MIE test.

## Discussion

The main aims of the present study were to (i) examine the effects of EIMD and muscle soreness on EIP and perception of effort during subsequent treadmill based MIE, (ii) explore the relationship between EIP, muscle soreness, EIMD and test duration in the MIE test, (iii) examine the effect of EIMD and muscle soreness on the physiological variables derived from treadmill MIE. The main findings in the present study were that a 30 min downhill run increased maximum EIP during MIE, and decreased TTE despite no change in ${\overset{\cdot }{\text{V}}\text{O}}_{2\text{max}}$ and RPE. Additionally, there was a significant association between EIP, MVC, and VA, with TTE during visit 6 (48 h post) (*p* ≤ 0.05) please see Table 4. Furthermore, there was a significant reduction in physiological variables obtained from the MIE test following the downhill run (e.g., decreased HR_max_). The significant (100%) increase in maximum EIP 48 h post, despite no change in ${\overset{\cdot }{\text{V}}\text{O}}_{2\text{max}}$ and RPE with concomitant decreases in TTE, BLa_max_ and HR_max_ following the downhill run suggests a “mismatch” between perceived effort and actual metabolic cost, resulting in the individual terminating the MIE test prematurely (Table 3).

TTE decreased on average by 34 s, 48 h post in EXP only, congruent with reports of a decrease in TTE during exercise to exhaustion (Davies et al., 2008, 2009, 2011; Doncaster and Twist, 2012; Chrismas et al., 2017), and distance covered during time-trials (Marcora and Bosio, 2007; Burt and Twist, 2011), following muscle damaging exercise. The test durations observed in the present study (Tables 1, 3) are considered valid for the determination of ${\overset{\cdot }{\text{V}}\text{O}}_{2\text{max}}$ testing (Midgley et al., 2008). The mechanisms underpinning the decrement in BLa_max_ are currently ambiguous within the literature; with reports of a decrease (Le Gallais et al., 1999), increase (Gleeson et al., 1998) and no change (Davies et al., 2011) during cycle ergometry based MIE under the presence of EIMD. The decrease in BLa_max_ observed in the present study may be due to preferential damage of type II (glycolitic) fibers during the downhill run, and therefore, a greater reliance on type I fibers 48 h post. However, it may also be due to a reduction in TTE as shown previously in treadmill based MIE. Similarly, the effects of muscle damaging exercise on HR_max_ during cycle ergometry (Le Gallais et al., 1999; Davies et al., 2011) and treadmill (Chrismas et al., 2017) based MIE are equally as inconsistent. Heart rate typically plateaus toward the end of MIE and HR_max_ may be achieved well before an individual terminates the test, which may help explain this finding. A higher ${\overset{\cdot }{\text{V}}\text{O}}_{2\text{max}}$ is typically observed in running compared to cycling due to the larger amount of muscle mass used, although this can be dependent on the characteristics of the population tested (Millet et al., 2009). However higher BLa_max_ may be expected following a cycling based MIE test due to the differences in energy metabolism between these two modalities (Millet et al., 2009). Furthermore HR_max_ has been reported to be approximately 5% higher in treadmill MIE tests compared to cycling MIE tests (Millet et al., 2009). Moreover, these differences between cycling and running, are also dependent on various confounding factors such as population demographics, laboratory environment, and protocol used, which makes the comparison between studies problematic. Nevertheless, as BLa_max_ and HR_max_ are commonly used as secondary criteria to establish if an individual has provided a maximal effort, if exercise tolerance following EIMD effects the “normal” BLa_max_ and HR_max_ response, verification of whether they provided a maximal effort could indeed be compromised, which would affect the accuracy of the results.

A higher percentage of participants in the EXP group reported pain as a major contributory factor 48 h post compared to CON. The increase in maximum EIP and decrease in physiological measures (i.e., BLa_max_ and HR_max_) during visit 6 (48 h post) arose without any change in RPE. Therefore, supporting the premise of a “mismatch” between perception of effort and actual metabolic cost following the downhill run. Once an individual has reached their tolerance for exercise (Ekkekakis et al., 2005) they will disengage from the task (Gibson et al., 2003). Previous research has suggested that EIP tolerance can predict cycling time trial performance (Astokorki and Mauger, 2016). Participants who had a higher EIP tolerance were able to produce faster time trials, and those with lower levels of EIP tolerance had slower time trial performance, therefore, demonstrating the importance of EIP tolerance. Subsequently, following a bout of muscle damaging exercise it appears individuals reach their tolerance for exercise quicker than they would do without any signs and symptoms of EIMD, and therefore, terminate the test (34 s decrease in TTE 48 h post in EXP - Table 3) prior to reaching their physiological maximum (determined in pre 1– Table 1). In support of these findings there was a significant relationship between TTE, and maximum EIP during visit 6 (48 h post) (*p* = 0.05) suggesting that maximum EIP can account for 8% (*R*^2^ value for maximum EIP) of the variance in TTE respectively (Table 4). The reasons for this premature termination of the test are likely due to the combination of noxious substance release (i.e., muscle soreness), and increased disruption to muscle fibers (i.e., impaired neuromuscular function). For the former, noxious substances released following the downhill run could subsequently stimulate group III/IV metabo-sensitive fibers, and provide afferent feedback to the motor cortex (Cheung et al., 2003), which in turn, may decrease central drive, and therefore, may be associated with decreased exercise duration (Amann et al., 2010). Additionally, afferent feedback from these receptors may also increase the conscious awareness of “discomfort,” in turn reducing the voluntary effort, ultimately resulting in an individual stopping the exercise (Hough, 1902). However, afferent feedback was not estimated in the present study, and therefore, caution must be taken when extrapolating the findings in this study in terms of afferent involvement of metabo-sensitive fibers. Additionally, as shown in Table 2, the CV for EIP is relatively high (23%), suggesting low reproducibility (Atkinson and Nevill, 1998). However, the average difference in maximum EIP between EXP and CON during visit 6 (48 h post) was 75%, demonstrating that the average minimum worthwhile difference (i.e., 75%) is significantly greater than the CV (23%).

The significant decrease in both MVC and VA in EXP visit 6 (48 h post) supports the notion that damage to the muscle fibers may have impaired neuromuscular function (i.e., peripheral fatigue), and decreased central drive (i.e., central fatigue). Damage to the structural proteins within the muscle fibers (i.e., decreased MVC) has been shown to lead to the perception of pain (Miller et al., 2004). Further indirect evidence for muscle fiber damage is supported by the significant increase in CK in the present study. CK does not typically leak out of undamaged cells (Lee and Clarkson, 2003), and therefore, an increase is primarily interpreted as an increased permeability or breakdown of the muscle cell membrane (Fridén and Lieber, 2001). Subsequently, increased CK efflux provides indirect evidence of ultrastructural damage from the downhill run (Allen et al., 1995). However, as shown in Table 2, the CV for CK is relatively high (20%), suggesting low reproducibility (Atkinson and Nevill, 1998). Subsequently, this measurement should not be used in isolation to determine the magnitude of EIMD. Nevertheless, as long as the CV of the measurement tool is smaller than the minimum worthwhile difference, the measurement tool can be deemed appropriate. The decrease in VA suggests that either there was a reduction in the number of motor neurons recruited, and/or they were firing at a sub-optimal rate (Behrens et al., 2012). The reduction in TTE was also significantly correlated with the decrease in both MVC and VA during visit 6 (48 h post) (*p* ≤ 0.05) supporting the premise that impaired neuromuscular function can account for 13% (*R*^2^ value for MVC), and 10% (*R*^2^ value for VA) of the variance in TTE respectively (Table 4). Ultrastructural damage to the muscle fibers as a consequence of the downhill run, may have increased muscle fiber recruitment in EXP during visit 6 (48 h post), in order to maintain the required exercise intensity (Eston et al., 2003). However, the likely preferential damage to type II fibers, may have resulted in the capacity to utilize only type I fibers, which due to their distinct physiological characteristics could account for the significant reduction in both MVC and BLa_max_.

## Conclusions

The findings in the present study demonstrate that a bout of downhill running induces the signs and symptoms of EIMD (i.e., muscle soreness), significantly increasing maximum EIP during a subsequent MIE test. Both EIP and impaired neuromuscular function as a result of this EIMD are associated with premature task disengagement during MIE (i.e., reduced TTE) despite no change in RPE. The exact mechanisms responsible for this mismatch between actual metabolic cost and perceived effort remain to be elucidated. It is presently not clear whether it is the release of noxious stimuli (i.e., EIP) and/or ultrastructural damage to muscle fibers that affect perception of effort. Future research should focus on revealing these mechanisms in order to understand the pain-perception nexus more clearly, and provide athletes and practitioners with appropriate pre-test procedures and recovery strategies to ensure MIE performance is not impaired. Based on the outcomes of the present study, it is suggested that > 48 h is required to ensure that muscle-damaging exercise does not reduce MIE test performance. However, further research should investigate the exact time frame necessary for complete recovery from muscle damaging exercise.

Perception of effort and EIP are clearly key components of not only test termination during MIE, but also fixed and self-paced exercise performance, and therefore, are an important area of concern for researchers, athletes and coaches. However, it is essential to consider that pain is a subjective emotion, and there may be large individual differences, particularly between athletes and non-athletes (i.e., those with prior experience), and the willingness to endure pain (Mauger, 2014). As the participants in the present study were only recreationally active they may have less willingness to continue to exercise whilst enduring pain, and therefore, may have terminated the test prematurely. Whether this relationship still exists in athletes during MIE following EIMD, is currently unknown (Mauger, 2014).

## Author contributions

All authors (BC, LT, JS, and AM) contributed to study conception, analysis and interpretation, drafting of the paper and final approval of the manuscript.

### Conflict of interest statement

The authors declare that the research was conducted in the absence of any commercial or financial relationships that could be construed as a potential conflict of interest. The reviewer AM and handling Editor declared their shared affiliation, and the handling Editor states that the process nevertheless met the standards of a fair and objective review.
